# Reliability and Validity of the Dutch Version of the Brief Infant-Toddler Social and Emotional Assessment (BITSEA)

**DOI:** 10.1371/journal.pone.0038762

**Published:** 2012-06-08

**Authors:** Ingrid Kruizinga, Wilma Jansen, Carolien L. de Haan, Jan van der Ende, Alice S. Carter, Hein Raat

**Affiliations:** 1 Department of Public Health, Erasmus University Medical Center, Rotterdam, The Netherlands; 2 Department of Youth Policy, Rotterdam Municipal Health Service (GGD Rotterdam-Rijnmond), Rotterdam, The Netherlands; 3 Department of Child and Adolescent Psychiatry, Erasmus University Medical Center-Sophia Children's Hospital, Rotterdam, The Netherlands; 4 Department of Psychology, University of Massachusetts, Boston, Massachusetts, United States of America; Tokyo Metropolitan Institute of Medical Science, Japan

## Abstract

**Background:**

The Brief Infant-Toddler Social and Emotional Assessment (BITSEA) is a relatively new and short (42-item) questionnaire that measures psychosocial problems in toddlers and consists of a Problem and a Competence scale. In this study the reliability and validity of the Dutch version of the BITSEA were examined for the whole group and for gender and ethnicity subgroups.

**Methods:**

Parents of 7140 two-year-old children were invited in the study, of which 3170 (44.4%) parents completed the BITSEA. For evaluation of the score distribution, the presence of floor/ceiling effects was determined. The internal consistency (Cronbach's alpha) was evaluated and in subsamples the test-retest, parent-childcare provider interrater reliability and concurrent validity with regard to the Child Behavioral Checklist (CBCL). Discriminative validity was evaluated by comparing scores of parents that worry and parents that do not worry about their child's development.

**Results:**

The BITSEA showed no floor or ceiling effects. Psychometric properties of the BITSEA Problem and Competence scale were respectively: Cronbach's alphas were 0.76 and 0.63. Test-retest correlations were 0.75 and 0.61. Interrater reliability correlations were 0.30 and 0.17. Concurrent validity was as hypothesised. The BITSEA was able to discriminate between parents that worry about their child and parents that do not worry. The psychometric properties of the BITSEA were comparable across gender and ethnic background.

**Conclusion:**

The results in this large-scale study of a diverse sample support the reliability and validity of the BITSEA Problem scale. The BITSEA Competence scale needs further study. The performance of the BITSEA appears to be similar in subgroups by gender and ethnic background.

## Introduction

Psychosocial problems, such as social-emotional and behavioural problems, are prevalent among 12% to 16% of two-year-old children [1]. Psychosocial problems in preschool aged children are associated with disorders later in life, such as oppositional defiant disorder, attention deficit disorder, conduct disorder, simple phobia, avoidant disorder and depressive disorder NOS [2], [3]. Measurement, early detection and treatment of psychosocial problems at a young age is important because this may contribute to a reduction of problems and an increase of competencies at older ages [4], [5]. To measure psychosocial problems, reliable and valid instruments are necessary.

Short comprehensive instruments that are appropriate to measure psychosocial problems in children of preschool age are limited [6]. Existing instruments, such as the Eyberg Child Behavior Inventory [7] or the Toddler Behavior Screening Inventory [8], only measure problem behaviour and do not address social-emotional competencies. Measuring delays in social-emotional competence, however, is also important since delays in competence are for instance related to internalising and externalising problems later in life [9]. There remains a need for a short instrument that measures both problems and delays in competence.

The Brief Infant-Toddler Social and Emotional Assessment (BITSEA) [10], developed in the United States of America, is a short (42-item) questionnaire measuring psychosocial problems and delays in the acquisition of competencies in toddlers. The BITSEA consists of a Problem scale and a Competence scale, and can be used in epidemiological studies, in (preventive) child health care and in early intervention settings for children between the ages of 12 and 36 months [10], [11]. The BITSEA is a shorter version of the Infant-Toddler Social and Emotional Assessment (ITSEA) [12], [13], which has been reported to have an acceptable factor structure, test-retest reliability, interrater reliability and validity in (a) community samples [13], (b) a sample of young children referred to an early intervention program [11] and (c) a clinical sample of young children referred for psychiatric assessment [14].

Only a few studies have evaluated the reliability and validity of the BITSEA [10], [15], [16]. The objective of this study was to investigate the following psychometric properties of the Dutch version of the BITSEA in a large sample of preschool children in the Netherlands:

1. the score distribution of the BITSEA;
2. the reliability of the BITSEA scale scores (internal consistency, test-retest reliability and interrater reliability);
3. the validity of the BITSEA scales interpretation (concurrent validity and discriminative validity).

Additionally we evaluated the score distribution, reliability and validity within subgroups of boys and girls, as well as native and immigrant children, because psychometric properties might differ between these subgroups [17], [18], [19].

## Methods

### Ethics statement

Part of the data became available in the context of the government approved routine health examinations of the preventive child health care. Separate informed consent was therefore not requested. Only anonymous data were used and the questionnaires were completed on a voluntary basis. Parents received written information on these questionnaires and were free to object to participation. Observational research with data does not fall within the ambit of the Dutch Act on research involving human subjects and does not require the approval of an ethics review board. As part of the data was anonymous for the researchers, this part of the study is not covered by the WMA Declaration of Helsinki. Informed consent was obtained for participation for the test-retest and interrater reliability data-collection, since these data were not anonymous and not part of the routine health examinations. This part of the study has been conducted according to the principles expressed in the WMA Declaration of Helsinki. The Medical Ethics Committee of the Erasmus Medical Centre Rotterdam approved the study protocol and consent procedures.

### Data collection

The present study was embedded in broader examinations of the BITSEA as an early detection tool of psychosocial problems in toddlers and has been described in detail elsewhere [20]. The present study was conducted in the larger Rotterdam area in the Netherlands among two-year-old children and their parents, who were invited between April 2010 and April 2011 by child health care organizations for well-child visits: A few weeks before the well-child visit was scheduled, parents of 7140 children received a child health monitor questionnaire by mail, including among others the BITSEA and Child Behavioral Checklist (CBCL1.5–5) and written information about the study. Parents decided for themselves whether the father or mother would complete the questionnaire. The parent-completed BITSEA was used by a child health professional during the well-child visit to assess the development of the child. Parents of 3320 (46.5%) children attended the well-child visit; 53.5% of invited parents did not attend the well-child visit and did not complete the questionnaire. Of those parents that did attend the well-child visit, 3170 (95.5%) handed in the completed child health monitor questionnaire. Children were excluded from the analyses if there were too many missing items (Problem scale >5, Competence scale >2) on both BITSEA scales (n = 43) [21], leaving a study population of 3127 (94.2%) children. The CBCL1.5–5 [22] was also included in the child health monitor questionnaire but only for research purposes (i.e. evaluating the concurrent validity of the BITSEA). Parents of 2304 (69.4%) children wanted to contribute to the study and also completed the CBCL1.5–5.

Test-retest and interrater reliability was evaluated in the subsample of parents that completed the child health monitor questionnaire in the month prior to receiving the questionnaire by the researchers. A subgroup of 314 parents were mailed the BITSEA again to assess the test-retest reliability which resulted in a response by parents of 120 (38.2%) children. The range of the period between completion of questionnaires was 13–77 days (*mean* = 44.7, *SD* = 18.1). Additionally, BITSEA questionnaires were mailed to childcare providers (i.e. child day care facilities outside home) of a subgroup of 130 children to assess interrater reliability, which resulted in a response of 75 (57.7%) completed questionnaires. The range of the period between completion of questionnaires was 3–76 days (*mean* = 45.8, *SD* = 21.5).

### Measures

The BITSEA consists of 42 items with three response options (‘not true/rarely’, ‘somewhat true/sometimes’, ‘very true/often’). Versions are available for parents and childcare providers. The childcare provider form is almost identical to the parent form but has some wording adaptations to make it appropriate for the childcare setting. The BITSEA is comprised of two multi-item scales, a Problem scale (31 items) and Competence scale (11 items), and responses can be summed for each scale. The possible score range of the Problem scale is 0–62 and of the Competence scale 0–22. A high score on the Problem scale or a low score on the Competence scale is less favourable [21]. In addition to the 42 items, the BITSEA has two single-item questions on parent worries regarding child language development and child behaviour, emotions or relationships. The BITSEA was translated into Dutch according to international guidelines [23].

In addition to the BITSEA, the CBCL1.5–5 was completed by parents in order to evaluate the concurrent validity of the BITSEA. The well-validated [22] 100-item CBCL1.5–5 is designed for children aged 18 months to 5 years and has two domains (Internalising and Externalising) and a Total Problem score. Answers are given on a 3-point scale (‘not true’, ‘somewhat or sometimes true’ and ‘very true or often true’).

Items on standard socio-demographic variables were included; which parent completed the questionnaire, ages of parents and child, child gender, child and parents' country of birth, parents' educational level and employment status, and family composition. A child was considered native if both parents were born in the Netherlands, a child was considered an immigrant if at least one of the parents was born outside the Netherlands [24].

### Analyses

Analyses were performed with SPSS 19.0 (SPSS Inc. 2010). Differences in mean BITSEA scores between boys and girls and between native and immigrant children were tested with independent sample t-tests.

#### Score distribution

Score distribution was evaluated by assessing the presence of floor and ceiling effects (i.e. >15% of the respondents have the minimal and/or maximal score) [25], mean scale scores and the 25^th^, 50^th^ and 75^th^ percentile points.

#### Reliability

Cronbach's alpha was used to evaluate the internal consistency of the Problem and Competence scales. An alpha of 0.70 or higher is considered acceptable [26]. Differences in internal consistency across gender and ethnic background subgroups was tested by computing critical F-statistics [27] with alpha set to 0.01. Test-retest and interrater reliability of the BITSEA-scales were assessed with the Intraclass Correlation Coefficients (ICC), using a two-way random effect model with absolute agreement. An ICC of 0.70 or higher is considered to indicate acceptable test-retest and interrater reliability [25]. To test the difference between gender and ethnic background subgroups for test-retest and interrater reliability, ICC Fisher r-to-z transformations were performed and a two-tailed criterion for significance was used.

#### Validity

Concurrent validity was evaluated by assessing Pearson correlations between BITSEA and CBCL1.5–5 scale scores. Concurrent validity is hypothesised to be expressed in large positive correlations and small to medium negative correlations between respectively BITSEA Problem and Competence scales with the CBCL1.5–5 Internalising, Externalising and Total Problem scores. A correlation of 0.1 is considered small, 0.3 is considered medium and >0.5 is considered large [28].

Discriminative validity is evaluated by assessing the ability of the BITSEA to discriminate between a subgroup *without* parents who reported worries about their child's behaviour, emotions or relationships and a subgroup *with* parents who reported worries about their child's behavior, emotions or relationships. This single-item question is part of the BITSEA, however does not add to either BITSEA scale score, therefore we regarded this question as suitable to evaluate discriminative validity. We hypothesised that discriminative validity will be reflected in less favourable BITSEA scores for children of parents with worries about their child [29]. Differences in mean BITSEA scores between these groups were tested with an independent sample t-test and effect sizes were defined as *d =* ***|*** *[mean(not worried)–mean(worried)]/SD(worried)* ***|***; [28] 0.20≤*d*<0.50 indicates a small effect, 0.50≤*d*<0.80 indicates a medium effect and *d≥*0.80 indicates a large effect. Discriminative validity, as described above, was also evaluated by gender and ethnic background subgroups. We hypothesised that we would find the same pattern of results within subgroups as in the general population.

## Results

Mean child age was 23.7 months (*SD* = 0.7), 48.9% were girls, and 55.7% of the children had a Dutch ethnic background. Mean age of the mother was 33.5 years (*SD* = 5.1) and mean age of the father was 36.3 years (*SD* = 5.5). In 88.1% of the cases the mother or both parents were the respondent(s). See Table 1 for more information on demographic characteristics of the study population.

**Table 1:** Characteristics of the study population, N = 3127.

	% of participants	Mean (SD)
***Mother characteristics***		
Age (years)		33.5 (5.1)
Country of birth (the Netherlands)	65.1	
*Educational level* 1		
-Lower general education or less	23.4	
-Intermediate vocational/pre-university	30.5	
-Higher vocational/university	39.6	
*Employment* 1		
-Employed	63.7	
-Homemaker	16.5	
-Unemployed	9.9	
***Father characteristics***		
Age (years)		36.3 (5.5)
Country of birth (the Netherlands)	61.4	
*Educational level* 1		
-Lower general secondary or less	21.6	
-Intermediate vocational/pre-university	27.7	
-Higher vocational/university	36.2	
*Employment* 1		
-Employed	79.3	
-Homemaker	0.8	
-Unemployed	6.6	
***Child characteristics***		
Age (months)		23.7 (0.7)
Gender (girls)	48.9	
Ethnic background^2^ (native)	55.7	
***Family characteristics***		
Two-parent household	82.5	
One-child family	42.1	
Respondent (mother or both parents)	88.1	

1 Percentages do not sum to 100 because of missing values.

2 A child is considered native when both parents were born in the Netherlands.

### Score distribution

Floor and ceiling effects were absent (Table 2). Mean scale scores and the 25^th^, 50^th^ and 75^th^ percentile points are presented in Table 2.

**Table 2:** Score distributions and internal consistency of BITSEA-scales, as reported by the parents, by gender and ethnic background, N = 2237.

BITSEA scales	Mean score (SD)	Range	% min^1^	% max^1^	25th %tile	50th %tile	75th %tile	Cronbach's alpha^2^
**Total** N = 2237								
Problem	7.8 (5.3)	0–40	1.8	0.0	4	7	10	0.76
Competence	17.5 (3.0)	0–22	0.1	5.6	16	18	20	0.63
**Boys** N = 1124								
Problem	8.2^a^ (5.6)	0–40	1.6	0.0	4	7	11	0.77
Competence	17.1^a^ (3.0)	1–22	0.0	4.0	15	17	19	0.61
**Girls** N = 1098								
Problem	7.4^a^ (4.9)	0–30	2.1	0.0	4	6	10	0.74
Competence	17.9^a^ (3.0)	0–22	0.1	7.4	16	18	20	0.65
**Native children** N = 1354								
Problem	6.7^b^ (4.4)	0–40	2.1	0.0	3	6	9	0.70
Competence	18.1^b^ (2.7)	4–22	0.0	7.6	16	19	20	0.60
**Immigrant children** N = 883								
Problem	9.3^b^ (5.9)	0–38	1.5	0.0	5	8	12	0.78
Competence	16.7^b^ (3.3)	0–22	0.1	3.1	15	17	19	0.64

1 % of respondents with the lowest (min) and highest (max) BITSEA scale score (ceiling/floor).

2 No significant differences between subgroups by gender or ethnic background in internal consistency, p>0.01

a = significant difference in mean BITSEA scores between boys and girls, p<0.01.

b = significant difference in mean BITSEA scores between native and immigrant children, p<0.01.

Boys had both a significantly higher mean Problem score (8.2, *SD* = 5.6) compared to girls (7.4, *SD* = 4.9), *p*<0.01, and a significantly lower mean Competence score (17.1, *SD* = 3.0) compared to girls (17.9, *SD* = 3.0), *p*<0.01. Immigrant children had both a significantly higher mean Problem score (9.3, *SD* = 5.9) compared to native children (6.7, *SD* = 4.4), p<0.01, and a significantly lower mean Competence score (16.7, *SD* = 3.3) compared to native children (18.1, *SD* = 2.7), *p*<0.01 (Table 2).

### Reliability

Internal consistency was 0.76 for the Problem scale and 0.63 for the Competence scale (Table 2). Test-retest reliability was 0.75 for the Problem scale and 0.61 for the Competence scale (Table 3). Parent/childcare provider interrater reliability was 0.30 for the Problem scale and 0.17 for the Competence scale (Table 3). No significant differences in reliability indices for gender and ethnic background subgroups were found.

**Table 3:** Test-retest and parent/childcare provider interrater reliability for BITSEA scores, by gender and ethnic background.

BITSEA scales		Problem			Competence		
		Test-retest reliability					
Sample	N	ICC	Mean (SD) test	Mean (SD) retest	ICC	Mean (SD) test	Mean (SD) retest
Total	120	0.75	7.1 (4.6)	6.6 (4.4)	0.61	18.1 (2.8)	18.1 (3.0)
Boys	63	0.70	7.5 (4.9)	7.0 (4.6)	0.66	17.4 (3.1)	17.3 (3.4)
Girls	57	0.82	6.6 (4.2)	6.2 (4.2)	0.41	18.8 (2.2)	19.1 (2.2)
Native children	87	0.66	6.4 (3.7)	6.2 (3.9)	0.57	18.3 (2.4)	18.5 (2.6)
Immigrant children	33	0.83	8.9 (6.0)	7.9 (5.4)	0.65	17.5 (3.5)	17.0 (3.6)

		Interrater reliability					
Sample	N	ICC	Mean (SD) parent	Mean (SD) childcare prov.	ICC	Mean (SD) parent	Mean (SD) childcare prov.
Total	75	0.30	7.3 (4.5)	7.5 (3.9)	0.17	18.6 (2.6)	16.1 (3.7)
Boys	38	0.34	7.1 (4.9)	7.4 (3.3)	0.19*	18.6 (2.3)	16.2 (3.3)
Girls	37	0.26*	7.5 (4.0)	7.6 (4.6)	0.16*	18.6 (2.9)	16.1 (4.2)
Native children	56	0.33	7.2 (4.6)	7.2 (4.1)	0.16*	18.8 (2.3)	16.4 (3.6)
Immigrant children	19	0.17*	7.5 (4.2)	8.3 (3.2)	0.15*	17.8 (3.3)	15.4 (4.2)

Note: There are no significant differences in ICC between gender and ethnic background.

* ICC is not significant, p>0.05.

### Validity

Concurrent validity: The BITSEA Problem scale was positively correlated with the CBCL1.5–5, Pearson coefficients of 0.66 (Internalising), 0.65 (Externalising) and 0.75 (Total Problem). The BITSEA Competence scale was negatively correlated with the CBCL1.5–5, Pearson coefficients of −.26 (Internalising), −0.23 (Externalising) and −0.26 (Total Problem). All correlations were significant, *p*<0.01. A similar pattern of correlations between BITSEA and CBCL1.5–5 was found for gender and ethnic background subgroups (Table 4).

**Table 4:** Concurrent validity (BITSEA and CBCL1.5–5) by gender and ethnic background, N = 2304.

Pearson correlation	Total		Boys		Girls		Native children		Immigrant children	
	Problem	Competence	Problem	Competence	Problem	Competence	Problem	Competence	Problem	Competence
**CBCL scales**										
Internalising	0.66	−0.26	0.65	−0.28	0.66	−0.24	0.60	−0.23	0.66	−0.20
Externalising	0.65	−0.23	0.67	−0.24	0.64	−0.21	0.64	−0.28	0.66	−0.13
Total Problems	0.75	−0.26	0.75	−0.29	0.75	−0.34	0.72	−0.27	0.75	−0.17

Note: All correlations are significant, p<0.01.

Discriminative validity: BITSEA scores of 482 (15.2%) children of parents who were worried were compared to BITSEA scores of 2621 (82.7%) children of parents that were not worried (percentages do not sum to 100% because of missing values). The mean BITSEA Problem score was higher in the ‘worried subgroup’ compared to the ‘not worried subgroup’, respectively *mean* = 12.8 (*SD* = 6.3) and *mean* = 6.9 (*SD* = 4.5), *p*<0.01, *effect size* = 0.93. BITSEA Competence scores were lower in the ‘worried subgroup’ compared to the ‘not worried subgroup’, respectively *mean* = 16.0 (*SD* = 3.5) and *mean* = 17.8 (*SD* = 2.8), *p*<0.01, *effect size* = 0.52. A similar pattern of differences in mean BITSEA scores between ‘worried’ parents and ‘not worried’ parents was found for gender and ethnic background subgroups (Table 5).

**Table 5:** Discriminative ability of the BITSEA between subgroups differing in parental worries about a child's behaviour, emotions or relationships, by gender and ethnic background, N = 3103.

Mean (SD)	Total			Boys			Girls			Native children			Immigrant children		
	Parental worries			Parental worries			Parental worries			Parental worries			Parental worries		
BITSEA scales	Not worried N = 2621	Worried N = 482	Effect size d^1^	Not worried N = 1301	Worried N = 253	Effect size d^1^	Not worried N = 1300	Worried N = 224	Effect size d^1^	Not worried N = 1574	Worried N = 173	Effect size d^1^	Not worried N = 1047	Worried N = 309	Effect size d^1^
Problem	6.9 (4.5)	12.8 (6.3)	0.93^a^	7.3 (4.7)	13.3 (6.9)	0.87 a	6.6 (4.3)	12.2 (5.5)	1.02^a^	6.2 (3.9)	11.4 (5.5)	0.95^a^	8.1 (5.1)	13.6 (6.6)	0.84^a^
Competence	17.8 (2.8)	16.0 (3.5)	0.52^b^	17.4 (2.9)	15.6 (3.4)	0.54^b^	18.2 (2.7)	16.5 (3.6)	0.48^c^	18.3 (2.6)	16.9 (3.1)	0.46^c^	17.1 (3.0)	15.5 (3.7)	0.44^c^

Note: All differences in means between ‘worried’ and ‘not worried’ are significant at p<0.01 for the total sample and subgroups.

1 Difference of the means divided by SD in the subgroup with worries.

a indicates a large effect (d≥0.8).

b indicates a medium effect (0.5≤d≥0.8).

c indicates a small effect (0.2≤d≥0.5).

## Discussion

The present study evaluated the psychometric properties of the Dutch version of the BITSEA in a large community sample in the Netherlands with a focus on differences across child gender and child ethnic background subgroups. The following psychometric properties of the BITSEA were determined in the present study: internal consistency, test-retest reliability, interrater reliability, concurrent validity and discriminative validity. The BITSEA Problem scale showed acceptable performance on all psychometric properties, whereas the BITSEA Competence scale showed acceptable performance on concurrent and discriminative validity. There were no differences in the psychometric properties of the BITSEA between boys and girls or between native and immigrant children.

### Score distribution

The BITSEA showed no floor or ceiling effects, which means that changes within toddlers with very low or very high scores can be measured. It also means that a toddler with a low score can be differentiated from other toddlers with low scores and that a toddler with a high score can be differentiated from other toddlers with high scores [25].

### Reliability

Internal consistency for the Problem scale was adequate (>0.70), but the internal consistency for the Competence scale was marginal (i.e. 0.63). Lower internal consistency for the Competence scale might be explained by inclusion of some items that assess behaviours that may not be expected to co-occur in young children, and items that are likely to show limited variability because they address early emerging competencies to identify significant social competence delays [10].

Test-retest reliability was adequate (>0.70) for the Problem scale and marginal (i.e. 0.61) for the Competence scale. These results mean that the BITSEA Problem scale provides stable outcomes over time, assuming that no real changes in psychosocial problems occur.

Interrater reliability was lower than the suggested guideline of 0.70. However, an interrater reliability meta-analysis of 119 studies, in which 26 studies reported interrater reliability between parent and teacher, found a mean correlation of 0.27 [30]. Correlations between parents and childcare provider/teacher are typically lower than correlations between parents. Lower correlations between measures of different observers can partly be explained by different settings in which a child is observed [30]. Compared to the mean reported parent-teacher interrater reliability, the Problem scale interrater reliability in this study was typical. However, the interrater reliability of the Competence scale was much lower than 0.27 and raises concerns about the reliability of this measure.

### Validity

As hypothesised, the BITSEA showed good concurrent validity; the BITSEA Problem scale had a strong positive correlation with CBCL1.5–5 Internalising, Externalising and Total Problem scores. Also as hypothesised, the BITSEA Competence scale had a negative correlation of medium strength with CBCL1.5–5 Internalising, Externalising and Total Problem scores.

The BITSEA scores were able to distinguish between parents reporting worry about their child's behaviour, emotions or relationships and parents who were not worried, indicating a good discriminative validity. Previous research illustrated a strong relationship between parents' concerns and children's developmental status [29], which supports our findings on the discriminative reliability of the BITSEA.

Mean BITSEA scores were less favourable for boys compared to girls, and for immigrant children compared to native children. These findings are in line with previous studies that report boys experience psychosocial problems more often than girls [31] and that psychosocial problems are more often reported in immigrant children compared to native children [32], [33].

The psychometric properties in this study are largely in line with what was found in previous studies on the BITSEA [10], [16]. One study found slightly higher internal consistency [16], another study found higher interrater reliability on the Competence scale and test-retest reliability [10] compared to our results. Differences in psychometric properties of the BITSEA may be explained by different social demographic characteristics and a different setting (e.g. in the other studies the BITSEA was not used by a child health professional to assess the child's development).

Our study has a few limitations. First, is that in the current study we have no data on the large non-response group. No information is available on parents that did not attend the well-child visit. It might be possible that parents avoid attending the well-child visit because they are afraid of possible interventions from Youth Care, but it might also be possible that parents do not find it necessary to attend the well-child visit because they feel confident that their child has no problems. Because the characteristics of the parents that are missed are unknown, it is unclear how the non-response has influenced the results on the psychometric properties of the BITSEA. However, we found no differences in psychometric properties within subgroups, so therefore we are confident that the non-response did not have a large impact on the outcomes. Second, the report by parents introduces the proxy-problem; self-report by two-year-old children on their psychosocial problems is not possible, because children of this age lack the necessary language skills and the cognitive abilities to interpret the questions and they do not have a long-term view of events [34]. Therefore, proxy by parents may be a useful alternative [35].

A major strength of our study is the large and diverse sample size. Additionally, the setting in which the respondents were invited to complete the BITSEA, the daily practice of well-child visit at the child health care centre, can be seen as either a strength or a limitation. We evaluated the psychometric properties in a setting in which the BITSEA might be implemented; however this specific setting might, on the other hand, hamper generalisations of our results to other settings.

We recommend future studies to evaluate the psychometric properties of the BITSEA in a different sample and setting. The setting in this study was the daily practice of a well-child visit in an urban area; but it would be good to be able to replicate these results in a more rural area, possibly outside the context of a well-child visit. Also, we recommend future studies to evaluate the BITSEA as an early detection tool for psychosocial problems in toddlers (i.e. the ability of the BITSEA to correctly classify children with and without psychosocial problems) for which the sensitivity and specificity of the BITSEA should be evaluated using a clinical sample of children with a diagnosis made by a professional [36]. Furthermore, referrals by child health professionals based on BITSEA scores and subsequent use of the (mental) health care system of children should also be investigated.

In conclusion, the results of our study support the reliability and validity of the BITSEA Problem scale. Further studies regarding the reliability of the Competence scale are advised. The performance of the BITSEA appears to be similar in boys and girls and in native and immigrant children. The BITSEA is a promising instrument to measure psychosocial problems in toddlers.
